# Supplementary material for: CXCL14 Attenuates Triple-Negative Breast Cancer Progression by Regulating Immune Profiles of the Tumor Microenvironment in a T Cell-Dependent Manner
Source: Int J Mol Sci. 2022 Aug 18;23(16):9314. doi: 10.3390/ijms23169314 (PMC9409254; doi:10.3390/ijms23169314)
Supplement: Supplementary file 1 [file ijms-23-09314-s001.zip › ijms-1817644-Supplementary Figure S2.pdf]

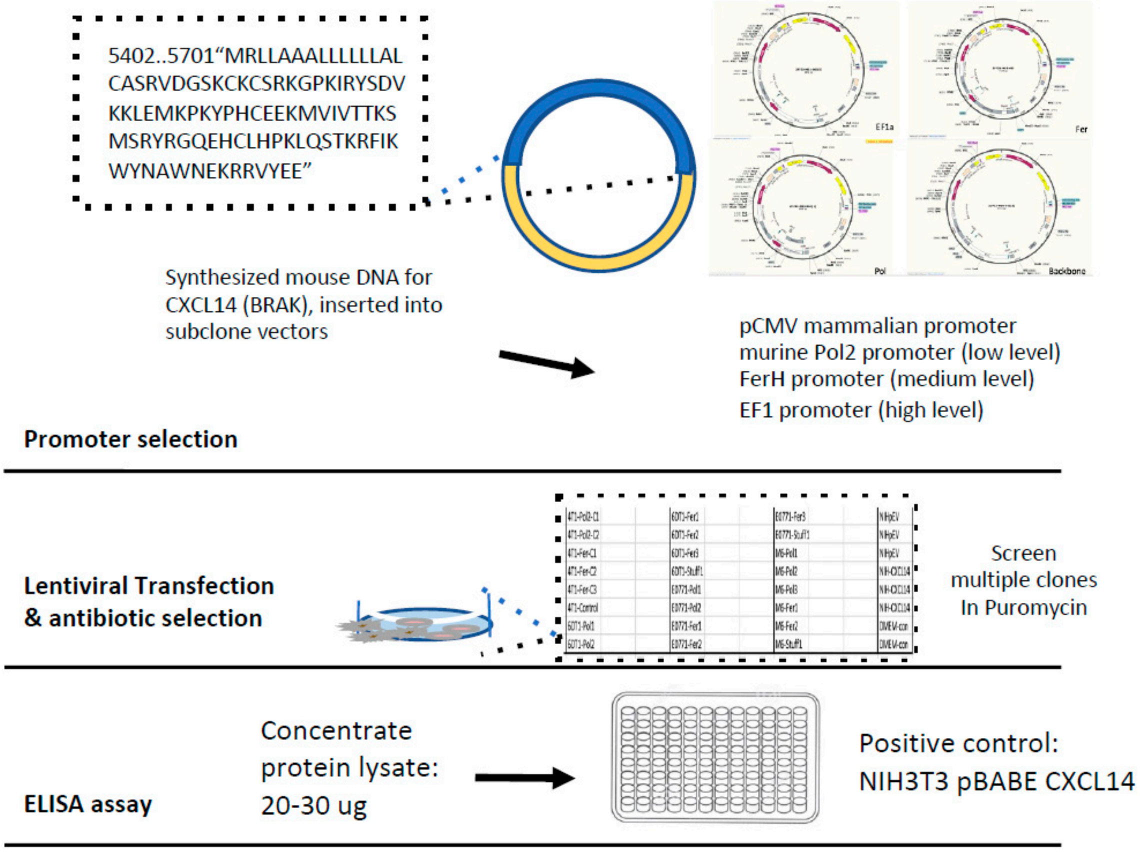

Supplementary Figure S2. Generation of CXCL14 over expressing 4T1 clonal cell lines. Constructs with different promoters were tested in lentiviral transduction experiments. A construct with FerH promoter containing, ampicillin resistant and a puromycin selection marker was selected. HEK293 cells were used to create viral particles. Particles from supernatant added to cells for 24 hours for viral transduction. Various mouse mammary breast cell lines were used. ELISA was performed in experimental triplicates.
