# Supplementary material for: CXCL14 Attenuates Triple-Negative Breast Cancer Progression by Regulating Immune Profiles of the Tumor Microenvironment in a T Cell-Dependent Manner
Source: Int J Mol Sci. 2022 Aug 18;23(16):9314. doi: 10.3390/ijms23169314 (PMC9409254; doi:10.3390/ijms23169314)
Supplement: Supplementary file 1 [file ijms-23-09314-s001.zip › ijms-1817644-Supplementary Figures.pdf]

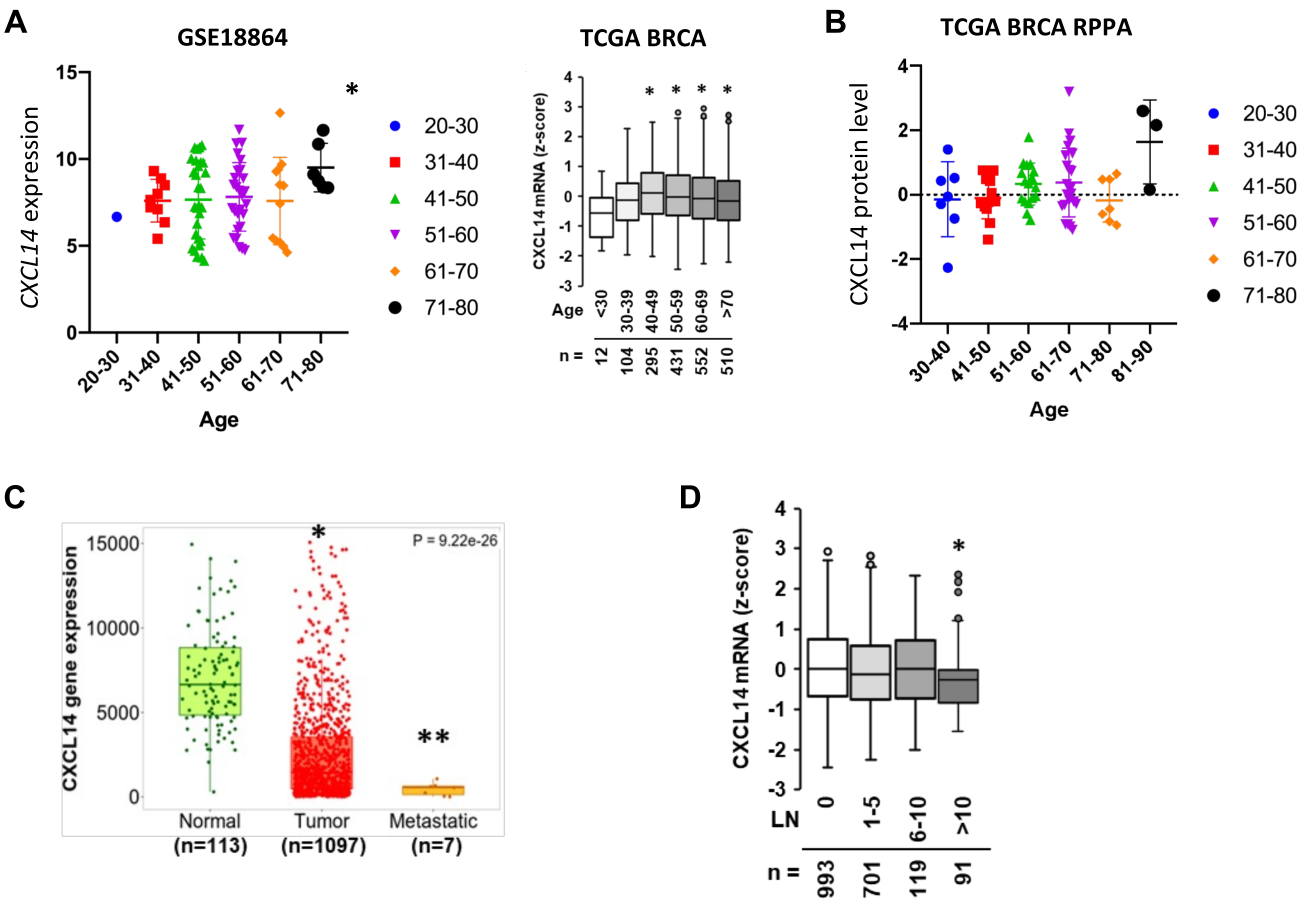

Supplementary Figure S1. Higher CXCL14 expression was found in older patients and its levels inversely correlated with breast cancer clinical characteristics.

(A) Correlation between CXCL14 expression levels and age of breast cancer patients. (B) Correlation between CXCL14 protein levels and age of breast cancer patients. (C) CXCL14 expression levels in normal tissue, primary tumors and metastatic tumors in TCGA breast cancer dataset. (D) Correlation between CXCL14 expression levels and number of lymph node metastases in TCGA breast cancer dataset.

A Surface markers for immune cells

FACS Panel	Antigen	Fluor	Species
Myeloid cells	CD11b	BB515	Rat IgG2b
	F4/80	PE	Rat IgG2a
	Viability	7AAD	
	CD45	PE-Cy7	Rat IgG2b
	Ly6C	AF700	Rat IgG2c
	Ly6G	APC	Rat IgG2a
	NK1.1	APC-Cy7	Mouse IgG2a
T/B cells	CD3	APC-Cy7	Hum IgG1
	CD19	AF700	Rat IgG2a
	CD4	APC	Rat IgG2a
	CD25	PE-Cy7	Rat IgG1
	viability	7AAD	
	CD8	FITC	Rat IgG2a
	CD45	PE	Mouse IgG1

B

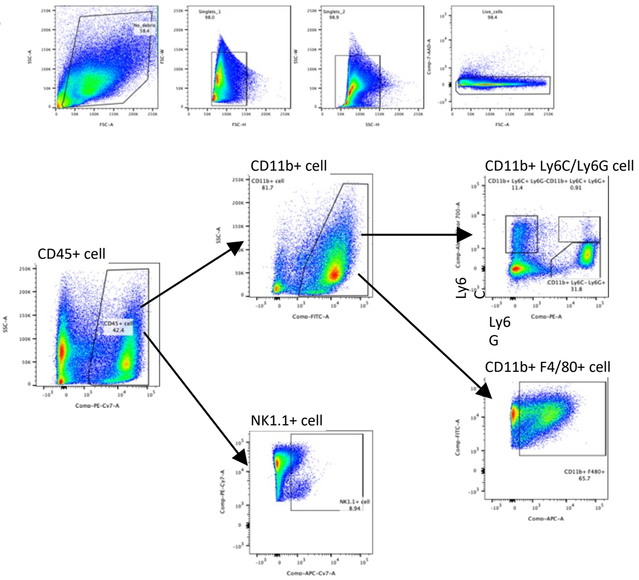

C

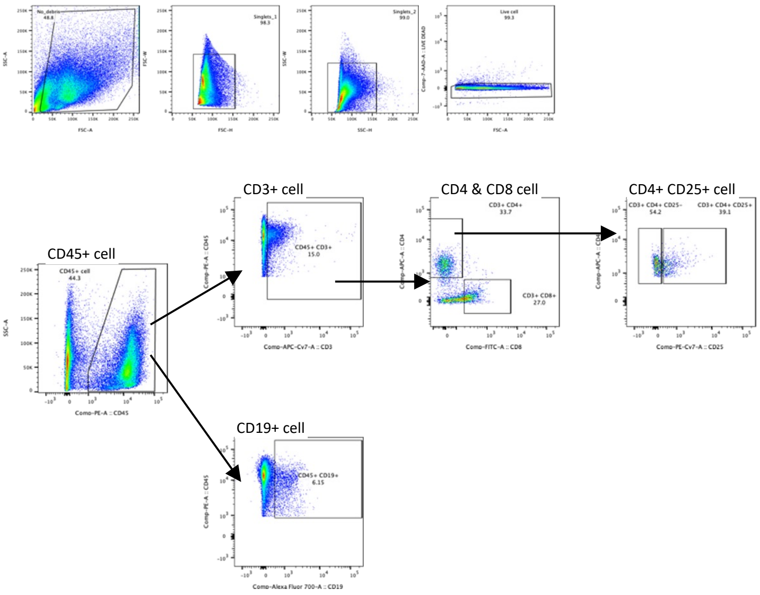

Supplementary Figure S3. Gating strategy for immune cell profiling of CXCL14 overexpression vs control. (A) Surface markers for immune profiling. (B) Flow cytometry gating strategy for myeloid cell panel. (C) Flow cytometry gating strategy for lymphoid cell panel.

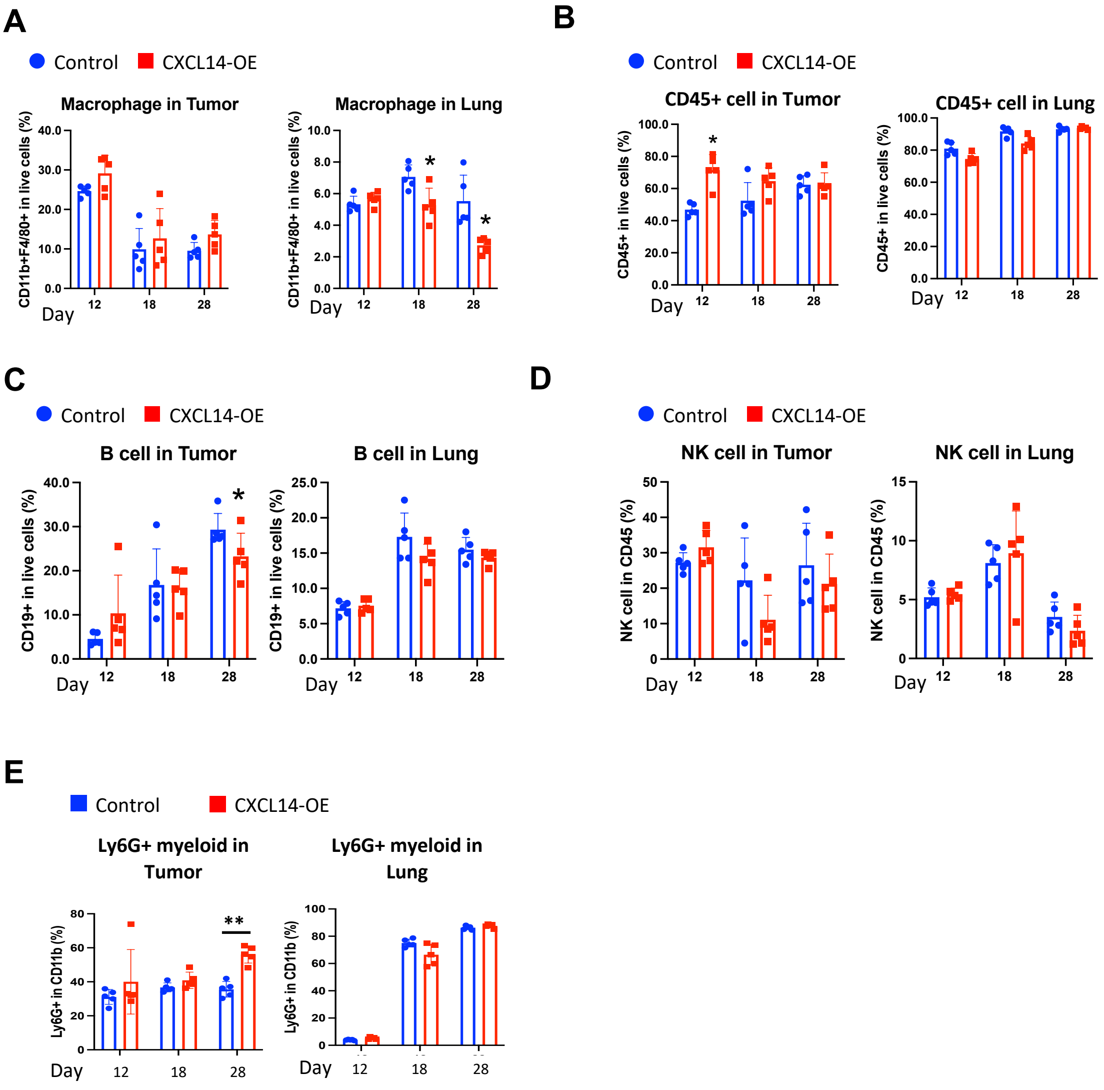

Supplementary Figure S4. CXCL14 overexpression and immune cell composition

(A) CXCL14 overexpression decreased macrophages in the lungs at day 18 and day 28, with no difference in the tumors. (B) CXCL14 overexpression did not change the number of CD45+ in the tumor or lung except an increase in CD45+ cells in day 12 tumors. (C) CXCL14 overexpression did not alter CD19+ B cell number in the lungs or day 12 and day 18 tumors except a decrease in CD19+ B cells in day 28 tumors. (D) No difference of NK cells by CXCL14 overexpression. (E) CXCL14 overexpression did not change the number of Ly6G+ cells in the lung or Day 12 and day 18 tumors except an increase in Ly6G+ cells in day 28 tumors.
